# The Role of Quorum Sensing in Phage Lifecycle Decision: A Switch Between Lytic and Lysogenic Pathways

**DOI:** 10.3390/v17030317

**Published:** 2025-02-26

**Authors:** Junjie Shang, Kehan Wang, Qian Zhou, Yunlin Wei

**Affiliations:** Faculty of Life Science and Technology, Kunming University of Science and Technology, Kunming 650500, China; sjjnjy117327@gmail.com (J.S.); 18087916582@163.com (K.W.); 17687026368@163.com (Q.Z.)

**Keywords:** phage, bacterium, communication, quorum sensing

## Abstract

Phages, the most abundant and diverse lifeforms on Earth, require strict parasitism for survival. During infection, temperate phages integrate both intracellular and extracellular host information to decide between lysis and lysogeny for replication. While various environmental and physiological factors influence the lysis–lysogeny decision, recent insights into phage–bacterium interactions reveal phages’ ability to communicate with and influence bacteria, leveraging the host’s quorum sensing system or small molecular signals. This article provides a succinct overview of current research advancements in this field, enhancing our understanding of phage–host dynamics and providing insights into bacteria’s multicellular behavior in antiviral defense.

## 1. Introduction

The evolutionary arms race between bacteria and phages has led to the development of sophisticated defense mechanisms in bacteria, such as restriction–modification (R–M) systems [1], CRISPR-Cas [2], toxin–antitoxin systems [3], and surface barriers [4]. These mechanisms not only highlight the survival strategies of these microorganisms but also provide valuable insights into microbial immunity. However, these interactions are further complicated by the ability of phages to sense and respond to bacterial population density through quorum sensing (QS). QS allows phages to adapt their infection strategy, switching between lytic and lysogenic cycles based on the density and behavior of bacterial hosts. This novel dimension of phage–bacteria interaction underscores the complexity of microbial defense and offers new avenues for therapeutic applications. Recent studies have illuminated the role of QS in modulating phage–host interactions. For instance, research on the phage VP882 revealed that it utilizes a QS system to influence its lysis–lysogeny decision, thereby affecting its replication strategy and potential to spread within the host population [5]. Additionally, a study on the lambda phage demonstrated that the CII protein, a key regulator in the phage’s life cycle, is influenced by environmental factors, including those affecting QS, which in turn impacts the phage’s decision to enter either the lytic or lysogenic pathway [6].

Understanding the interplay between QS and phage infection cycles is crucial for developing more effective phage therapies. By integrating QS mechanisms into phage therapy, we can potentially enhance phage efficacy, control bacterial populations more precisely, and mitigate the development of bacterial resistance. This approach not only deepens our knowledge of microbial immunity but also opens the door to more refined and effective strategies for managing bacterial infections, particularly in the face of rising antimicrobial resistance [7].

The R–M system acts by recognizing and cleaving foreign DNA while protecting the bacterial genome through methylation [8]. The CRISPR–Cas system, on the other hand, provides adaptive immunity by capturing and storing phage genetic material, enabling precise defense against subsequent infections with the same phage [9]. Toxin–antitoxin systems regulate the release of cellular toxins, aiding in the inhibition of phage replication, while defense islands, with their unique gene clusters, offer additional protection [10]. Additionally, bacteria can alter surface structures or induce self-destruction of infected cells to prevent phage adsorption and propagation [11].

With the advancement of research on phage–bacterial interactions, it has become evident that phages exploit host bacterial quorum sensing systems or small molecule signals to communicate with bacteria and influence their behavior. Recent studies have utilized a range of experimental techniques to detect and manipulate these quorum-sensing signals. For example, acyl-homoserine lactones (AHLs), a major class of signaling molecules in Gram-negative bacteria, are commonly quantified using high-performance liquid chromatography (HPLC) or mass spectrometry (MS) to assess their concentration in bacterial cultures. These methods enable researchers to measure the accumulation of AHLs in real time, providing insight into how phages may respond to bacterial population density. Additionally, genetic analyses, such as the use of reporter constructs with *luxR* or other QS receptor genes, have been employed to identify specific signaling molecules and their interactions with phage receptors. These genetic tools allow for precise manipulation of bacterial quorum sensing pathways, offering a way to dissect the role of QS in modulating phage decision-making between lytic and lysogenic cycles. For instance, studies have shown that introducing QS inhibitors or disrupting QS pathways in bacterial strains can significantly alter the behavior of infecting phages, supporting the notion that QS is a key regulator of phage–host dynamics. Research by Liu et al. and Gutiérrez et al. further illustrate the use of AHL quantification and genetic tools to explore phage responses to quorum sensing and to identify how these molecules impact the efficiency and timing of phage infection [12]. This review will explore these interactions from the perspective of QS, providing insights into the mechanisms by which phages may manipulate bacterial communication and behavior. It is hoped that this discussion will contribute to a deeper understanding of these complex interactions and guide future research in this area.

### 1.1. Definition and Classification of Phages

Phages, viruses that specifically infect bacteria, are estimated to number over 10^31^ in the biosphere [13]. They account for 20–40% of bacterial mortality daily [14], significantly influencing Earth’s biogeochemical cycles. This vast interaction underscores their role in shaping microbial populations and the planet’s ecological balance [14,15,16].

**Figure 1:**
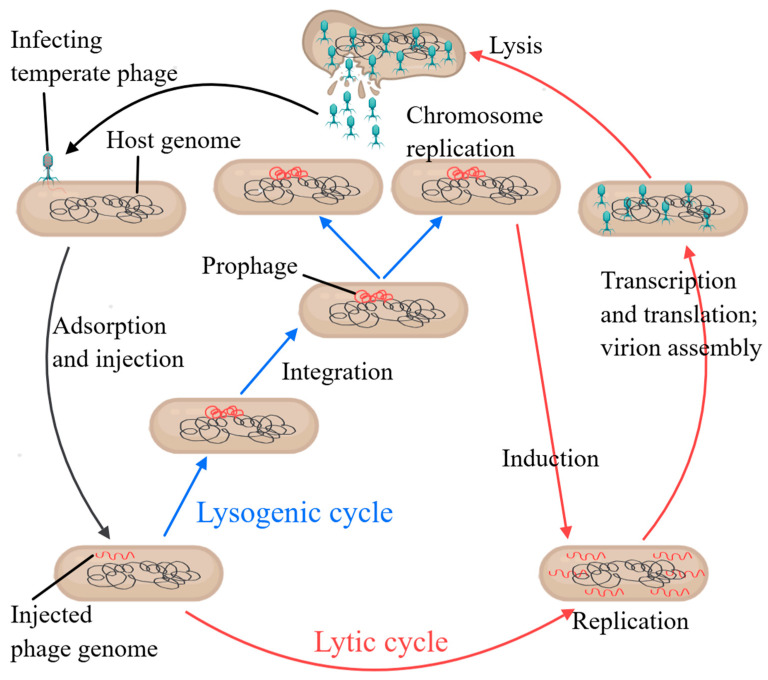
Lytic and lysogenic life cycles of phages [20]. General model of phage lytic and lysogenic life cycles. Following binding to specific host cell receptors, temperate phages inject their DNA (red) into the bacterial cell. Once injected, the phage genome can undergo either a lytic or a lysogenic cycle. During the lytic cycle, the phage genome replicates using host cell machinery, synthesizing phage proteins to produce mature virus progeny. Finally, lysis of the bacterial cell releases new virions that then infect other bacterial cells. Conversely, during lysogeny, the phage genome is integrated into the host chromosome (prophage) and replicates passively during host cell division. Prophage induction can occur following certain environmental stresses, where the phage genome is excised from the host chromosome and enters into the lytic cycle.

Phages are commonly classified into two categories based on their reproductive strategies: (1) lytic phages, which hijack the host bacterial machinery to replicate their genomes and produce capsid proteins, subsequently releasing progeny by inducing host cell lysis via the action of holins and endolysins; and (2) temperate phages, which integrate their genetic material into the bacterial genome and replicate alongside the host cell as a prophage. This integration confers resistance to subsequent infections by similar phages and may also impart new physiological traits to the host, establishing a symbiotic relationship. While a single infection typically triggers a lytic cycle, multiple infections often result in lysogeny [17]. Notably, the lytic and lysogenic cycles are interconnected, with temperate phages capable of switching between these two states in response to environmental cues or changes in host bacterial density, which are detected through quorum-sensing molecules [18]. Upon infection, the fate of a bacterial cell is determined by the interplay between viral decision-making and the bacterial defense system. In most cases, the phage’s decision predominates, dictating whether the cell undergoes lysis or is maintained as a host. Under these circumstances, the bacterial cell has little control over its fate. However, in certain bacteria that have evolved specialized defense mechanisms, infection can be actively halted upon detection. For instance, bacterial cells equipped with the abortive infection (Abi) system can terminate their own viability, thereby preventing further phage propagation and protecting the surrounding bacterial population [19] (Figure 1).

The lysis–lysogeny decision in phages is regulated by specific molecular mechanisms, exemplified by the temperate phage λ in *Escherichia coli.* This system has long been used as a model for understanding phage decision-making because of its well-characterized genetic framework and the ability to switch between lytic and lysogenic cycles in response to environmental cues [21]. The choice of *E. coli* and phage λ is not only based on their simplicity and tractability in laboratory settings but also on their ecological and clinical relevance. *E. coli* is a key component of the human gut microbiota and is often associated with pathogenic strains that cause a wide range of infections, from urinary tract infections to sepsis [22]. The ability of phage λ to regulate its lifecycle in response to environmental conditions is ecologically significant, as lysogeny can facilitate phage persistence in bacterial populations, potentially leading to horizontal gene transfer and the spread of virulence factors. Moreover, phage therapy, particularly targeting *E. coli* in clinical settings, has gained attention as an alternative to antibiotics in the face of rising antibiotic resistance [23]. This makes the *E. coli*–phage λ system particularly relevant for both basic research and therapeutic applications.

The lysis–lysogeny decision is primarily influenced by the expression of regulatory genes during the early stages of infection. In the λ phage system, environmental factors such as low temperature, high multiplicity of infection (MOI), reduced cell size, and nutrient scarcity promote lysogeny. Conversely, conditions that favor optimal viral replication tend to bias the decision toward lysis [24,25,26].

In the lysis–lysogeny decision of bacteriophage λ, several key regulators, including CI, CII, CIII, N, Cro, Q, PL, PR, and the integrase (Int) enzyme, play crucial roles in determining whether the phage enters the lytic or lysogenic cycle. When CI protein levels are high, CI binds to the lysogenic promoters (PL and PR), repressing the expression of lytic genes and thereby directing the phage toward the lysogenic cycle, where it stably maintains its genome within the host. CII is a critical activator that promotes the maintenance of lysogeny by enhancing CI expression, facilitating entry into the lysogenic cycle [27]. CIII further stabilizes CII by protecting it from degradation by host proteases, ensuring that CII can effectively activate CI expression and support lysogenic commitment. Without the protective function of CIII, CII would be rapidly degraded, shifting the decision in favor of the lytic cycle. N is an antitermination factor that plays a pivotal role in early phage gene expression by preventing premature transcription termination, allowing the continuation of gene expression. N is essential for initiating the lytic cycle by enhancing transcriptional efficiency and supporting the synthesis of lytic proteins. Similarly, Q functions as another antitermination factor but primarily acts in the late stages of the lytic cycle. It prevents transcription termination signals from halting gene expression, ensuring the full activation of lytic genes necessary for phage replication and host cell lysis. Cro acts as an antagonist to CI, promoting the lytic cycle by inhibiting CI expression. When Cro levels are high, it competitively binds to the lysogenic promoters, repressing CI synthesis and ultimately triggering the lytic cycle. PL and PR are two major phage promoters that regulate early gene transcription. PL drives the expression of lytic genes, while PR is responsible for the transcription of lysogeny-associated genes. CI maintains the lysogenic state by binding to PL and PR, suppressing their activity and preventing lytic gene expression. The Int enzyme is a key factor in the lysogenic cycle, facilitating the integration of the phage genome into the host bacterial chromosome. This integration ensures the stable maintenance of the prophage within the bacterial genome, allowing the phage to persist in the lysogenic state [12].

At the early stage of infection, the concentrations of CII and CIII are low, allowing Cro to competitively inhibit CI and activate the expression of lytic genes. N and Q further enhance the transcription of lytic genes, promoting the progression of the lytic cycle. As the infection progresses, the concentrations of CII and CIII gradually increase. Once a critical threshold is reached, CI is activated, which in turn represses the activity of PL and PR, thereby maintaining the lysogenic state and preventing the expression of lytic genes. CII and CIII collectively support lysogeny by promoting CI expression and inhibiting the function of Cro. The molecular basis of this decision lies in the interplay between the CII protein, N anti-terminator protein, and Q anti-terminator protein. This interaction subsequently regulates the expression of the CI protein and the Int gene, which are critical for determining the phage’s fate between lysis and lysogeny [24]. Increased levels of CII promote lysogeny by inhibiting the expression of the lysis gene Cro and facilitating the activation of Int, which enables the integration of phage DNA into the bacterial genome. Conversely, reduced CII levels lead to the activation of Cro expression, which is repressed by the CI repressor protein, thereby favoring the lytic cycle [28]. Subsequently, the function of the Q anti-terminator facilitates the expression of structural and lysis genes. The expressed proteins will then assemble new phage particles and lyse the host cell, thereby initiating the lytic cycle through the activation of lysis genes mediated by the Q anti-terminator [29].

Elevated levels of CII promote lysogeny by inhibiting the expression of the lytic gene Cro and facilitating the activation of Int, which drives the integration of phage DNA into the bacterial genome. In contrast, reduced CII levels result in the derepression of Cro expression, which is normally inhibited by the CI repressor protein, thereby tipping the balance towards the lytic cycle [30]. Additionally, various environmental and physiological factors can influence the phage’s decision to enter either the lytic or lysogenic cycle. These factors include fluctuations in salinity, aeration, nutrient availability, temperature, pH, and exposure to antibiotics, as well as external stimuli such as ultraviolet light, hydrogen peroxide, pollutants, and changes in bacterial or phage density. Furthermore, interactions with other prophages may also play a role in modulating this decision [24,31,32,33,34,35,36,37,38,39,40].

**Figure 2:**
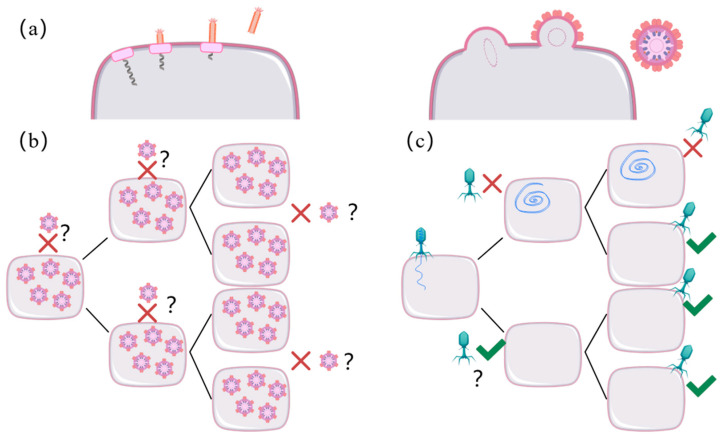
Chronic infection and pseudolysogeny of bacteriophages [42]. (**a**) Productive, chronic infection in which progeny phage particles are released by extrusion (left) or by budding (right) through the cell membrane without lysing the host bacterium. (**b**) Non-productive, chronic infection, in which large amounts of intracellular phage particles are produced without host lysis. The intracellular phage particles may confer superinfection exclusion. (**c**) Pseudolysogeny displays a stalled phage development stage in which the unintegrated phage genome is asymmetrically passed on to daughter cells. Daughter cells may become resistant (indicated by red crosses) to secondary infections through the inheritance of the phage genome or, as in the case of phage P22, immunity factors [43]. Upon the dilution of the immunity factors through subsequent cell divisions, the resistant subpopulation ultimately becomes sensitive to phage infections (indicated by green ticks).

In nature, phages employ additional infection strategies beyond the conventional lytic and lysogenic cycles, including pseudolysogeny and chronic infection. Pseudolysogeny, a less common phenomenon induced by nutrient deprivation, involves the phage genome persisting as a dormant, non-integrated entity within the host. This state is maintained until conditions improve, at which point the phage may either enter the lytic cycle or establish lysogeny [24]. In contrast, during chronic infection, phages replicate continuously within the host and are released gradually without inducing cell lysis. This process allows for the sustained release of viral particles over an extended period [41] (Figure 2). It should be noted that not all phages are capable of pseudolysogeny—indeed, pseudolysogeny is a variant form of lysogeny—whereas the strictly virulent phages we discuss are lytic by nature, a trait determined by the phage’s own genetic makeup and mechanisms.

### 1.2. Introduction of Quorum Sensing

Recent decades of research have significantly broadened our understanding of communication, extending it beyond multicellular organisms to encompass microorganisms. Bacteria, for example, utilize small molecules to communicate both with each other and with eukaryotic hosts, thereby influencing a range of physiological processes, from gene expression to cell interactions. A key example of microbial communication is QS, in which bacteria assess their population density through the secretion of autoinducers (AIs) and use this information to coordinate collective behaviors such as biofilm formation, antibiotic resistance, and host interactions. Although QS systems and their specific responses can vary across species, the fundamental principles underlying these systems are highly conserved. At low cell densities, AI concentrations are insufficient to activate QS; however, as bacterial populations increase, the accumulation of AIs triggers coordinated actions that benefit the community, such as bioluminescence or the expression of virulence factors [44,45,46]. Additionally, QS is counterbalanced by quorum quenching (QQ), which disrupts these communications [47]. Intriguingly, recent research shows that phages can employ a similar arbitration system to gauge their densities, influencing their decision between lytic and lysogenic cycles [48,49,50]. This review highlights significant advances in understanding how quorum sensing mediates phage–host interactions.

## 2. Quorum Sensing in the Interaction Between Phages and Hosts

### 2.1. Information Transfer Between Phage and Host

Lysogeny is considered a strategic adaptation that enables phages to survive under unfavorable conditions, such as nutrient scarcity, which may reduce host availability and hinder the production of viral progeny. The effectiveness of this strategy is often inversely related to ecosystem productivity, as low-nutrient environments typically limit host density, thereby promoting lysogeny over lysis [51]. AHLs are signaling molecules commonly used by Gram-negative bacteria in quorum sensing, a mechanism through which bacteria communicate and coordinate their behavior based on population density. The ability of bacteria to detect and respond to AHLs allows them to synchronize behavior for survival and adaptation in complex environments. In the context of bacteriophage biology, AHL-mediated quorum sensing plays a critical role in determining the decision between lytic and lysogenic cycles. Phages, through the detection of AHLs, can adjust their replication strategy to either rapidly kill the host (lytic) or integrate into the host genome and persist (lysogenic), depending on the environmental conditions and the bacterial population’s signaling [52]. This theory is supported by observations that high bacterial densities can trigger prophage induction, while experiments with AHL in soil and groundwater have shown increases in viral populations [53,54]. These insights highlight bacterial density as a critical factor in phage–host interactions, suggesting that phages evaluate host density through various quorum-sensing signals. This understanding enhances our knowledge of how phages make developmental decisions, implicating quorum sensing systems as key elements in their decision-making processes. It is important to note that viruses themselves do not possess the characteristics of being “alive” and rely entirely on their hosts for essential processes such as transcription and translation. Here, we refer to the phenomenon whereby viruses manipulate host-encoded receptors to competitively detect signal molecules from the bacterial community. When the stimulatory signal from these molecules reaches a critical threshold, the phage switches from lysogeny to lysis. Moreover, because the reception of these signals is competitive, phages are inherently less efficient than their bacterial hosts in assessing population density, and their response is temporally delayed [55].

Research has revealed complex interactions between phages and bacterial QS networks. Gosh et al. demonstrated that AHLs or AHL-producing strains could trigger SOS-independent induction of prophage λ in *E. coli* [56,57,58,59], a process mediated by the *E. coli* Sensing of autoinducers A (SdiA) receptor, which typically does not produce AHLs [59]. This induction is influenced by SdiA and Regulator of Chromosome Segregation A (RcsA), both associated with extracellular polysaccharide synthesis and implicated in λ prophage induction, with AHL enhancing RcsA activity [59]. To validate the role of QS in this process, appropriate control experiments should involve the use of quorum-sensing-deficient bacterial strains or QS inhibitors. For example, QS-deficient *E. coli* strains lacking *sdiA* or *rcsA* could be tested for prophage λ induction in response to AHLs. Similarly, the use of QS inhibitors, such as synthetic AHL analogs or natural QS inhibitors, could further confirm the specific signaling pathway mediating prophage induction [60].

Taj et al. discovered that indole, another signal molecule, can mitigate T4 phage infection in *E. coli* via QS [51,56]. Control experiments using indole-deficient *E. coli* strains or indole inhibitors could help establish a direct link between indole signaling and phage interference. Additional studies identified mechanisms in other bacteria: prophage T1 in *E. coli* ATCC 15144 responds to cell-free supernatant signals, triggering lysogenic to lytic shifts [51,57], and similar QS-dependent prophage inductions are noted in *Enterococcus faecalis*, *Vibrio cholerae*, and *Pseudomonas aeruginosa* [61,62,63].

However, research on *Vibrio anguillarum* indicates an inverse relationship at high cell densities, where QS inhibits prophage induction, highlighting the nuanced roles of QS components like Vancomycin resistance protein O (VanO) and Vancomycin resistance protein T (VanT) in this process [64,65,66,67]. In addition, QS inhibitors in *P. aeruginosa* can facilitate lytic phage infections, underscoring the interplay between phage dynamics and bacterial QS systems [68,69]. These studies emphasize the necessity of control experiments to verify the role of QS signals, including the use of QS-deficient strains and specific inhibitors, to distinguish between QS-mediated and non-QS-mediated effects on phage dynamics.

Recent studies highlight the role of the *Pseudomonas* quinolone signal D (PqsD) enzyme, a critical component of the Pseudomonas Quinolone Signal (PQS) biosynthesis pathway, in modulating phage–host dynamics. For instance, the strictly lytic phage LUZ19 exhibits reduced infectivity in *P. aeruginosa* PqsD-deficient strains, but this defect is rescued by exogenous supplementation of HHQ (2-heptyl-4-hydroxyquinoline), a PQS precursor [70,71,72]. This suggests that PQS signaling may act as a bacterial defense mechanism by either attenuating phage-induced lysis or restricting viral propagation within bacterial communities. Notably, infection with the PQS-associated phage DMS3vir activates host PQS signaling, which suppresses bacterial swarming motility and promotes the formation of isolated, metabolically active bacterial clusters [68,73]. Conversely, adaptive studies with phage PT7 indicate that PQS signaling can enhance bacterial resistance to phage predation, underscoring the dual role of QS in phage–bacterial interactions [61,70]. These findings align with broader observations that bacterial QS systems, such as the *las* and *rhl* systems in *P. aeruginosa*, dynamically regulate phage receptor expression and biofilm formation, thereby influencing phage infectivity and host susceptibility [64,74,75].

**Figure 3:**
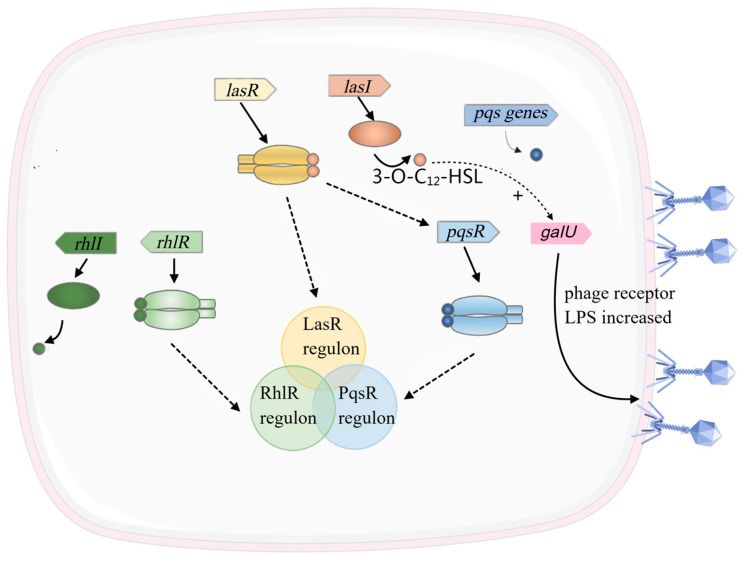
*Pseudomonas aeruginosa* quorum sensing [74]. LasR is one of the core regulatory factors of the QS system in *P. aeruginosa*, belonging to the *Las* system, with its signaling molecule being 3-O-C_12_-HSL. The LasR regulon controls the expression of a series of key virulence factors and genes related to biofilm formation [74]. The *las* QS positively regulates the expression of *galU*, which is involved in LPS biosynthesis, thereby promoting phage adsorption [81]. Rhl regulator (RhlR) is another key regulatory factor in the QS system of *P. aeruginosa*, belonging to the Rhl system, with its signaling molecule being C_4_-HSL. The RhlR regulon mainly regulates genes involved in biofilm formation, motility, and virulence [82]. The regulator of quorum sensing regulated by the signal molecule (PqsR) is another QS transcription factor in *P. aeruginosa* that participates in the regulation of genes associated with various physiological processes [83]. The coordination of these QS systems enables *P. aeruginosa* to adjust its pathogenicity, virulence factor secretion, antibiotic resistance, and biofilm formation behaviors according to population density and environmental conditions.

In contrast, LUZ19 appears to elicit a milder stress response, possibly exploiting PQS signaling to enhance its own infective success without prompting strong defensive reactions [70,72,76]. Conversely, adaptive experiments with phage PT7 suggest that PQS signaling might bolster bacterial resistance to phages [77]. Furthermore, experiments using attenuated phages have demonstrated that selective pressure can induce mutations in key quorum-sensing regulators, such as *lasR* and *mvfR*, thereby disrupting essential QS pathways, including AHL and PQS (Figure 3). These mutations may alter the host’s susceptibility to phage infections, potentially enhancing or mitigating phage attack efficiency [78,79,80].

### 2.2. Utilization of QS and QQ Receptor Homologs by Phages

**Figure 4:**
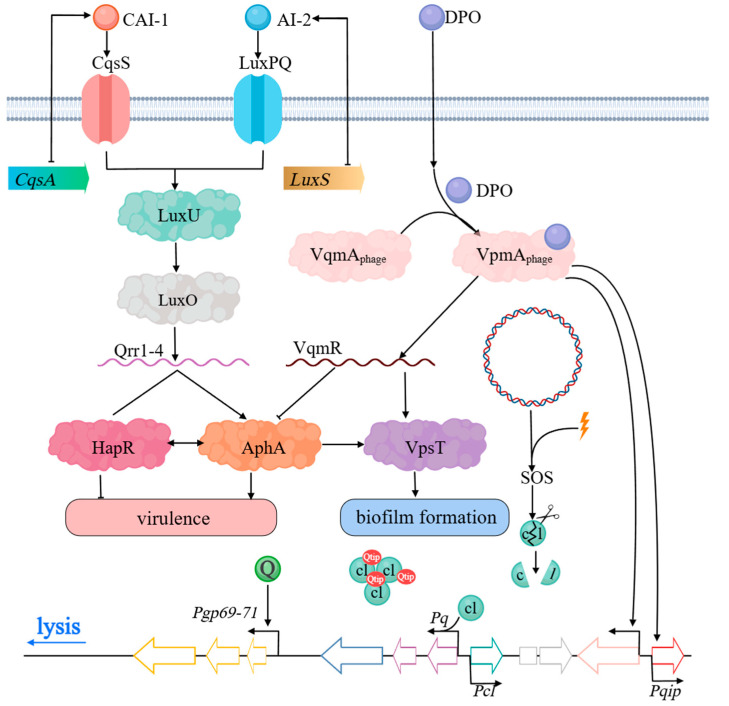
*Vibrio cholerae* quorum sensing [88]. The quorum sensing system of *V. cholerae* is initiated by two primary autoinducers, CAI-1 and AI-2, along with their respective receptors, CqsS and LuxPQ. Through the intermediary signal transduction molecules LuxU and LuxO, the production of Qrr sRNAs is regulated, thereby balancing the expression of AphA at low cell densities and HapR at high cell densities. Simultaneously, DPO modulates the key regulator VpsT of biofilm formation via the VqmA/VqmR branch, further refining the quorum sensing network’s control over bacterial collective behavior. This system enables *V. cholerae* to flexibly regulate virulence and communal behaviors in response to variations in cell density and environmental conditions, thereby providing a strategic advantage for its survival and dissemination both within and outside the host [88,89]. Phage VP882 (multi-colored strips) can lyse or lysogenize its *Vibrio* host. In the lysogenic state, Q (green) production is repressed by cI (light green). Lysis depends on the inactivation of cI activity, and that is mediated by two independent inputs, host DNA damage or QS. Host DNA damage (lightning bolt) leads to RecA-assisted proteolysis (scissors) of the cI repressor. The QS input is mediated by VqmA_Phage_ (pink) binding to the host-produced DPO AI, which is derived from threonine via the Tdh enzyme. VqmA_Phage_ bound to DPO (purple) activates the expression of *qtip* (red). Qtip aggregates the cI protein. Irrespective of the input, reduced cI activity leads to the derepression of *q* and subsequent expression of genes involved in the lytic cycle (blue). VqmA_Phage_, when bound to host DPO, also activates transcription of the host VqmA QS target, *vqmR*, leading to the production of the sRNA VqmR. The VqmR regulon includes genes required for biofilm formation [78].

Recent research has illuminated the molecular mechanisms by which QS influences the lytic-lysogenic switch in phages, particularly through phage-encoded QS homologs that can manipulate or alter host cellular processes. Silpe and Bassler elucidated a mechanism in which the phage VP882 detects the signaling molecule 3,5-Dimethylpyrazin-2-ol (DPO) via its own VqmA receptor, a homolog of the Multiple Virulence Factor Regulator [78]. This interaction triggers a shift in the phage life cycle, promoting the transition to the lytic phase [84] (Figure 4). In *Vibrio cholerae*, DPO engagement with bacterial VqmA activates *Vibrio* quorum-sensing regulatory RNA (VqmR) expression, an sRNA that suppresses biofilm and toxin gene expression [85]. Phage VP882 capitalizes on this system, using DPO-bound VqmA to trigger its *qtip* gene expression, which subsequently inhibits CI, leading to lytic gene activation [84,86]. Notably, this interaction exhibits an asymmetric dynamic: while the phage-encoded VqmA receptor can recognize and respond to host QS signals, the bacterial VqmA does not interact with phage-specific promoters. This unidirectional influence enables phage VP882 to exploit host QS signaling to regulate its reproductive strategy, demonstrating a sophisticated level of control over its life cycle and interactions with the host [84,87].

Recent metagenomic and genomic explorations have unveiled novel QS-related genes in phages, such as those in *Clostridium tyrobutyricum* phage φCTP1, which targets *Aeromonas spp.*, and others bearing Accessory gene regulator (*agr*) system homologs (*agrB*, *agrC*, and *agrD*) in phages infecting *Bacillus spp.* and *Clostridium difficile* [90]. The absence of the response regulator AgrA in these phages suggests they might utilize the host’s Accessory gene regulator A (AgrA) or alternative effectors for signal transduction [90]. Additionally, phages infecting *Pseudomonas spp.* have been found to encode Lytic Transcriptional Regulator (LytTR) domain proteins, potentially acting as AgrA homologs to facilitate QS response regulation [91]. Intriguingly, phylogenetic analyses suggest that phage-encoded *agr* homologs, like those in phiCDHM1, likely originated from host bacteria through horizontal gene transfer, particularly from *Clostridium* strains’ *agr3* locus [90,92,93]. To investigate the functionality of QS-related phage proteins, Silpe and Bassler expressed recombinant LuxR_φARM81ld_ and LuxR_Apop_ receptors [85], revealing their insolubility in lysates but solubility upon binding specific AHL signal molecules (C4-HSL, C6-HSL, and C8-HSL) [85]. Although these LuxR receptors demonstrate AHL binding capability, their precise roles in the phage lifecycle and interplay with host QS systems remain to be fully elucidated [90,94,95].

Leblanc et al. identified phage ϕPLPEa, which infects purple bacteria and noted its unique acyl hydrolase gene, indicative of a relationship with bacterial quorum sensing [96]. Despite its predominantly virulent nature, ϕPLPEa retains a repressor homolog, suggesting it may represent a virulent phage that has acquired a regulatory gene or a temperate phage that has lost most lysogeny-associated genes. Of particular interest is the presence of an acyl hydrolase, an enzyme rarely found in phage genomes, which specifically targets homoserine lactone signal molecules. This unique capability may enable ϕPLPEa to disrupt or modulate bacterial QS, thereby influencing bacterial communication. Such modulation could confer competitive advantages to the phage’s host or alter interactions among neighboring bacterial populations, potentially affecting both bacterial behavior and the phage’s lytic-lysogenic decision-making processes [96,97,98]. Such a function underscores the sophisticated interplay between phages and bacterial quorum sensing, opening new avenues for understanding phage roles in microbial ecosystems.

### 2.3. Arbitrium System: A New Phage–Phage Communication System

Small molecule signaling is integral to the regulation of bacterial physiology, influencing processes such as transcription, metabolism, and cellular interactions. A central aspect of this signaling is QS, a communication mechanism that enables bacteria to coordinate collective behaviors in response to population density. This is achieved through the secretion and detection of specific signaling molecules known as autoinducers, which allow bacteria to sense the presence and concentration of neighboring cells and adjust their activities accordingly.

A newly identified peptide-based communication mechanism, termed the ’arbitration system’, has been found in phages phi3T and SPbeta [99]. This system comprises three key components: *aimP*, which encodes a precursor peptide processed into a mature signaling peptide upon extracellular protease action; *aim Regulator* (*aimR*), which encodes a receptor possessing a tetrapeptide repeat (TPR) domain characteristic of the Rap, Rgg, NprR, PlcR and PapR (RRNPP) family, commonly associated with Gram-positive bacteria’s quorum sensing; and *aim regulator X* (*aimX*), responsible for producing a regulatory RNA molecule and a small non-coding RNA that inhibits lysogeny [100,101]. Initial studies on phage Wbeta suggest that this non-coding AimX RNA can downregulate the lysogenic regulator *cI* gene through antisense RNA interactions [93]. This system underscores a sophisticated level of inter-phage communication, potentially influencing the phage’s lifecycle decisions.

**Figure 5:**
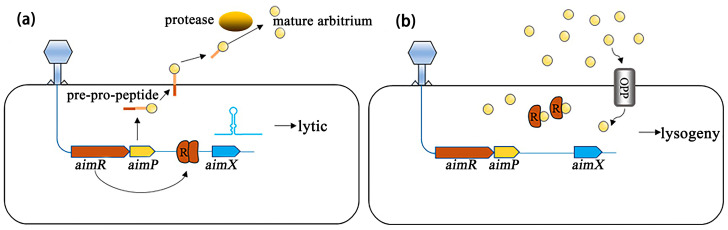
Mechanistic model for arbitrium system [50]. (**a**) At the first encounter of a phage with a bacterial population, the early genes *aimR* and *aimP* are expressed immediately upon infection. AimR, as a dimer, activates AimX expression. AimX is an inhibitor of lysogeny, possibly as a regulatory ncRNA, directing the phage to a lytic cycle. At the same time, AimP is expressed, secreted, and processed extracellularly to produce the mature peptide. (**b**) At later stages of the infection dynamics, the arbitrium peptide accumulates in the medium and is internalized into the bacteria by the OPP transporter. Now, when the phage infects the bacterium, the expressed AimR receptor binds the arbitrium molecules and cannot activate the expression of AimX, leading to lysogeny preference.

Upon infecting a host, phage phi3T synthesizes both the Aim regulator P (AimP) hexapeptide and the AimR receptor [99]. Initially, AimP levels are too low to interact with AimR, allowing the AimR dimer to bind upstream of the *aimX* gene and activate lytic gene expression (Figure 5a) [50]. As the infection cycle progresses and more phi3T particles infect cells, AimP accumulates in the environment and is taken up by adjacent bacterial cells via the oligopeptide permease (OPP) system [50]. When AimP internalizes and interacts with AimR, it triggers a structural shift in the receptor, breaking the dimer into inactive monomers. These monomers no longer bind to the *aimX* promoter, shifting the phage towards lysogeny (Figure 5b) [50]. In the case of SPbeta, the binding of AimP to AimR impedes the complex’s ability to recognize and bind the *aimX* operon [102], thus facilitating the transition to a lysogenic state [103].

Bioinformatics studies have revealed that the arbitration system might be a prevalent mechanism facilitating the co-evolution of phages and conjugation elements within the Firmicutes phylum. Erez et al. discovered 112 *aimR* homologs in the SPbeta group phages, with *aimP* and *aimX* homologs identified in 72% and 15% of these cases, respectively, predominantly within the *aimR-aimP-aimX* cluster [48]. A broader analysis across bacterial, archaeal, and viral genomes highlighted 1180 *aimR* homologs in phages, especially within SPbeta and Wbeta groups, and conjugation factors, with *aimP*-like genes accompanying 96% of them [93]. The AimR homologs diversified into 10 evolutionary branches, exhibiting varied peptide sequences and lengths, aligning with the diversity seen in the RRNPP receptor family [104,105]. Despite their phylogenetic distinction from RRNPP members, structural comparisons of SPbeta AimR suggested enough functional parallels to consider classifying AimR as a new member of this receptor family [102,103,106].

The arbitration peptides EIKPGG and MMSEPGGGGW, originating from the Wbeta and Waukesha92-like phage groups, respectively, have been shown to induce lytic activity when introduced at a concentration of 1 μmol/L, confirming their roles in phage arbitration systems [107]. Structural investigations reveal that these arbitration peptides share a conserved C-terminal region, although only a few residues critical for selectivity have been pinpointed within the AimR binding site [103,106,108]. The observed promiscuity of the AimR receptor, capable of interacting with various similar AimP peptides, suggests potential crosstalk among akin phages, potentially enhancing their adaptive capabilities [103,109]. Moreover, the discovery of AimR receptors across different phages and conjugation factors, capable of recognizing each other even in the absence of their own peptide signals [104], underscores a complex communication network that may significantly influence phage dynamics and host interactions.

These investigations reveal that phages possess population regulation mechanisms akin to those of bacteria, employing QS to optimize their infective strategies [39]. From the perspective of a phage, exploiting QS provides a strategic advantage for more effective host invasion by adapting its reproductive strategy in response to fluctuations in host density. Conversely, for bacteria, QS-regulated anti-phage defenses represent a sophisticated survival mechanism. As bacterial density increases, the risk of phage predation also rises. In response, bacteria can fine-tune their defense strategies by activating QS-controlled anti-phage pathways, thereby enhancing their ability to resist phage attacks with greater precision [1]. Such interplay underscores a sophisticated microbial arms race, where both phages and bacteria dynamically adjust their strategies in response to population density cues.

### 2.4. Specificity and Evolutionary Dynamics of QS in Phage–Host Interactions

The specificity of bacterial QS systems in modulating phage behavior raises critical questions about whether all bacterial QS signals are strictly phage-type specific and how phages evolved to exploit these communication networks. While QS systems are highly conserved across bacterial species, their molecular components—including autoinducers, receptors, and regulatory circuits—exhibit significant diversity, often tailored to ecological niches or host physiology. This diversity suggests that phages have co-evolved with their bacterial hosts to recognize and respond to specific QS signals, enabling precise adaptation to host population dynamics. However, recent studies indicate that some phages can respond to broadly conserved QS signals, such as AHLs, rather than strictly species-specific QS pathways [90,96].

For instance, phage VP882 specifically detects DPO via its VqmA receptor, a homolog of the host’s QS regulator, to initiate lysis in *V. cholerae* [78,87]. Similarly, *P. aeruginosa* phages such as LUZ19 and DMS3vir interact with PQS pathways, which are unique to this bacterial genus [67,70]. These examples highlight that many phages target QS components exclusive to their host’s taxonomic group, implying evolutionary specialization. The molecular basis for this specificity lies in the structural compatibility between phage-encoded receptors (e.g., LuxR-type proteins) and host-derived autoinducers, shaped by prolonged host-phage co-evolution [85,90]. However, some phages can recognize QS molecules used by multiple bacterial species, suggesting that QS exploitation may not always be strictly host-specific [92,110].

The evolutionary origins of QS exploitation by phages remain debated. One hypothesis posits that phage-encoded QS homologs, such as VqmA or LuxR-like receptors, were acquired through horizontal gene transfer (HGT) from bacterial hosts [90,96]. Genomic analyses of Clostridium phages and SPbeta-like phages support this, revealing *agr* system homologs (*agrB*, *agrC*) with high similarity to host genes [92,110]. Such HGT events would allow phages to “eavesdrop” on host communication systems, integrating these modules into their regulatory networks to optimize infection strategies. Comparative genomics of QS-associated phage genes suggests that certain phages may have acquired QS receptor homologs multiple times independently, further supporting an HGT-driven model of evolution [39,104].

Alternatively, convergent evolution may explain the emergence of phage-encoded QS-like systems, where selective pressures favored the independent development of signal-sensing mechanisms to counter host defenses [39,104]. Experimental studies have shown that phages infecting *P. aeruginosa* exhibit mutations in QS-related genes under prolonged co-culture conditions, potentially reflecting adaptive responses to host QS regulation [68,74]. Furthermore, structural analysis of phage-encoded LuxR homologs suggests functional divergence from bacterial counterparts, implying de novo evolution of QS sensing capabilities in some phages [85,90].

Notably, the arbitrium system—a peptide-based communication network in Bacillus phages—exemplifies an evolutionary innovation distinct from bacterial QS. Unlike bacterial autoinducers, arbitrium peptides (e.g., AimP) are phage-encoded, enabling inter-phage coordination to regulate lysogeny in response to viral population density [39,48]. Structural studies indicate that AimR, the receptor for arbitrium peptides, exhibits similarities to RRNPP family quorum-sensing regulators in bacteria, suggesting a shared evolutionary origin [39,104]. This system likely evolved to prevent premature host depletion, balancing phage fitness with ecological stability [39,111]. The coexistence of both host-derived QS exploitation and phage-specific communication systems underscores the dynamic interplay of adaptation strategies in phage evolution.

The selective advantages of QS specificity are manifold. For bacteria, QS-mediated defenses, such as biofilm formation or receptor downregulation, can deter phage adsorption [68,74]. In response, phages may evolve enhanced specificity to circumvent these defenses, driving an evolutionary arms race. For example, *P. aeruginosa LasR* mutants resistant to phage infection exhibit reduced expression of QS-controlled receptors, yet phages like vB_Pae_PLY counter-adapt by exploiting alternative QS-regulated surface molecules [68,74]. Such reciprocal adaptations highlight the role of frequency-dependent selection in shaping QS-phage interactions [1,70].

In summary, the specificity of QS in phage–host interactions reflects a complex tapestry of co-evolutionary adaptations. Phages exploit host QS systems through molecular mimicry or HGT-acquired components while also evolving novel communication strategies like arbitrium to navigate ecological challenges. Future studies integrating comparative genomics, experimental evolution, and structural biology will further elucidate how these systems emerged and diversified, offering insights into the evolutionary plasticity of microbial interactions.

## 3. Conclusions

The burgeoning study of QS in phages sparks several intriguing inquiries: Can phages discern different QS signals to selectively infect bacteria equipped with those systems? Might phages evolve to harness additional QS or QQ homologs, thereby manipulating bacterial communications to their advantage? Are there other unique communication systems, akin to the arbitration system, employed by phages [112]? Furthermore, with multiple signal molecules at play, it remains to be clarified how these can act as either promoters or inhibitors of phage replication and whether various communication mechanisms might be activated concurrently or interactively [112,113,114], fostering competition or cooperation. While the QS dynamics within bacterial communities are relatively well-elucidated [115,116], understanding how phages interact with these systems is still an emerging area of research [91]. Recent studies have highlighted how phages can detect bacterial QS signals and modulate their infection strategies accordingly [48,59,78]. The current study adds to this body of work by investigating how phages utilize QS systems to control their decision between lytic and lysogenic cycles. While previous research has confirmed that QS-regulated prophage induction is a widespread phenomenon [59], current research provides new insights into the specific molecular interactions between QS signals and phage regulators, revealing potential mechanisms that remain underexplored. For example, the role of AHLs in regulating the prophage λ induction in *E. coli* via SdiA receptors is consistent with earlier work by Ghosh et al. [59]. But adds a novel layer by linking extracellular polysaccharide synthesis to phage induction. Furthermore, this study highlights the potential of bioinformatics approaches to predict QS-controlled mechanisms in phage–host interactions, an area that has received limited attention in previous studies. By leveraging computational tools to identify QS signatures and their interaction with phage genomic elements, we open new avenues for exploring the co-evolutionary dynamics between phages and bacteria. Overall, while the foundational concepts of QS in phage–host communication are well-supported by prior studies, the current findings provide new insights into the specific mechanisms at play and offer a refined understanding of microbial ecosystem dynamics and diversity [116].

The exploration of communication mechanisms between phages and bacteria is crucial for leveraging these interactions in environmental and medical contexts. By manipulating signal molecules and phages, it is possible to control the populations of detrimental bacteria, aiding in pollution mitigation and ecosystem management [111,117]. Moreover, in an era of escalating antibiotic resistance, phage therapy emerges as a promising alternative, potentially circumventing the challenges posed by resistant bacterial strains [118,119]. A nuanced comprehension of phage–bacterial communication can enhance the predictability, efficacy, and safety of phage therapy. For example, stimulation of the quorum sensing system in *P. aeruginosa* upregulates the expression of genes associated with phage receptors, thereby enhancing phage adsorption and infectivity. Additionally, the *lasR* gene promotes the synthesis of lipopolysaccharides (LPS) and type IV pili, which further increases the infectivity of phage vB_Pae_PLY. Moreover, the phage-encoded quorum sensing system can be modulated by natural and synthetic inhibitors, offering promising avenues for the development of novel antibacterial strategies [81,120]. Strategically combining signal molecules with phages might not only counteract bacterial defenses but also amplify the therapeutic potential of phages against pathogenic bacteria, offering innovative solutions to combat antibiotic-resistant infections.

QS provides a comprehensive framework for understanding the intricate interactions between phages and bacteria, influencing their behaviors and physiological responses. Recent advancements in QS research hold the potential to significantly enhance our understanding of phage–host dynamics, offering deeper insights into the complexity of microbial ecosystems. This regulatory mechanism is crucial for optimizing phage survival and replication while reducing the risk of host extinction. However, the significance of these findings extends far beyond phage–host interactions. Understanding the role of quorum sensing in phage decision-making provides deeper insights into microbial ecosystem dynamics. In particular, it offers a valuable perspective on how microbial populations—including phages, bacteria, and other microorganisms—maintain stability, resilience, and diversity under fluctuating environmental conditions [93].

The QS mechanisms discovered in temperate phages have profound implications for understanding microbial ecosystem dynamics and diversity. QS enables phages to “sense” the density and behavior of their bacterial hosts and adjust their life cycle accordingly, switching between lysis and lysogeny. This process not only influences individual phage–host interactions but also shapes broader ecological equilibria within microbial communities. By regulating phage replication, QS helps control bacterial population size, preventing the overgrowth of certain strains while allowing others to thrive, thereby promoting biodiversity. This regulation is particularly critical for maintaining the stability of microbial ecosystems, where different populations interact and depend on each other for survival in natural environments [39].

In the context of environmental changes, such as nutrient availability or the presence of antimicrobial agents, QS-mediated decision-making can enhance microbial resilience. By opting for lysogeny under unfavorable conditions, phages can remain dormant, safeguarding their genetic material until conditions improve. This mechanism not only supports phage survival but also influences the evolutionary trajectory of their bacterial hosts, fostering genetic diversity and the potential for adaptive traits. Studies by Avelino Alvarez-Ordóñez et al. and Diana P. Pires et al. on QS in phage–host interactions have highlighted how these molecular signaling networks contribute to microbial community structure and resilience, underscoring the intricate balance that governs microbial ecosystems [121,122]. The interplay between QS, phage infection cycles, and microbial diversity presents an exciting avenue for further exploration of ecological dynamics, with implications not only for natural ecosystems but also for biotechnological applications such as phage therapy.

This expanded understanding enriches our knowledge of microbial ecology, revealing that interactions between viruses and their hosts are not merely competitive but rather part of a finely tuned regulatory system that sustains microbial diversity across various habitats [123].

Such knowledge could facilitate the development of novel preventive and therapeutic strategies against bacterial infections. By unraveling how phages exploit or disrupt bacterial QS, researchers can create innovative methods to modulate bacterial behavior, combat antibiotic resistance, and improve the effectiveness of phage therapy. Future studies in this area are expected to unveil transformative applications in microbiology, infectious disease management, and other related fields.

## Data Availability

The data that support the findings of this study are included within the article.

## References

[1] Wang Y.R., Fan H.H., Tong Y.G. (2023). Unveil the Secret of the Bacteria and Phage Arms Race. Int. J. Mol. Sci..

[2] Barrangou R., Fremaux C., Deveau H., Richards M., Boyaval P., Moineau S., Romero D.A., Horvath P. (2007). CRISPR provides acquired resistance against viruses in prokaryotes. Science.

[3] LeRoux M., Laub M.T. (2022). Toxin-Antitoxin Systems as Phage Defense Elements. Annu. Rev. Microbiol..

[4] Moller A.G., Lindsay J.A., Read T.D. (2019). Determinants of Phage Host Range in *Staphylococcus* Species. Appl. Environ. Microbiol..

[5] Shivam S., Li G.L., Lucia-Sanz A., Weitz J.S. (2022). Timescales modulate optimal lysis-lysogeny decision switches and near-term phage reproduction. Virus Evol..

[6] Shotland Y., Shifrin A., Ziv T., Teff D., Koby S., Kobiler O., Oppenheim A.B. (2000). Proteolysis of bacteriophage λ CII by *Escherichia coli* FtsH (HflB). J. Bacteriol..

[7] Cao L., Mi J.H., He Y.L., Xuan G.H., Wang J.X., Li M.Z., Tong Y.G. (2024). Quorum sensing inhibits phage infection by regulating biofilm formation of *P. aeruginosa* PAO1. J. Virol..

[8] Loenen W.A.M., Dryden D.T.F., Raleigh E.A., Wilson G.G., Murray N.E. (2014). Highlights of the DNA cutters: A short history of the restriction enzymes. Nucleic Acids Res..

[9] Jackson S.A., McKenzie R.E., Fagerlund R.D., Kieper S.N., Fineran P.C., Brouns S.J.J. (2017). CRISPR-Cas: Adapting to change. Science.

[10] LeRoux M., Srikant S., Teodoro G.I.C., Zhang T., Littlehale M.L., Boron S., Badiee M., Leung A.K.L., Sorek R., Laub M.T. (2022). The DarTG toxin-antitoxin system provides phage defence by ADP-ribosylating viral DNA. Nat. Microbiol.

[11] Chapot-Chartier M.-P. (2014). Interactions of the cell-wall glycopolymers of lactic acid bacteria with their bacteriophages. Front. Microbiol..

[12] Kobiler O., Rokney A., Friedman N., Court D.L., Stavans J., Oppenheim A.B. (2005). Quantitative kinetic analysis of the bacteriophage λ genetic network. Proc. Natl. Acad. Sci. USA.

[13] Los M., Wegrzyn G., Lobocka M., Szybalski W.T. (2012). Pseudolysogeny. Advances in Virus Research.

[14] Suttle C.A. (2007). Marine viruses—Major players in the global ecosystem. Nat. Rev. Microbiol..

[15] Hurwitz B.L., Hallam S.J., Sullivan M.B. (2013). Metabolic reprogramming by viruses in the sunlit and dark ocean. Genome Biol..

[16] Ji X.L., Zhang C.J., Fang Y., Zhang Q., Lin L.B., Tang B., Wei Y.L. (2015). Isolation and characterization of glacier VMY22, a novel lytic cold-active bacteriophage of *Bacillus cereus*. Virol. Sin..

[17] Hobbs Z., Abedon S.T. (2016). Diversity of phage infection types and associated terminology: The problem with ‘Lytic or lysogenic’. Fems Microbiol. Lett..

[18] Court D.L., Oppertheim A.B., Adhya S.L. (2007). A new look at bacteriophage λ genetic networks. J. Bacteriol..

[19] Lopatina A., Tal N., Sorek R., Enquist L. (2020). Abortive Infection: Bacterial Suicide as an Antiviral Immune Strategy. Annual Review of Virology.

[20] Brady A., Felipe-Ruiz A., del Sol F.G., Marina A., Quiles-Puchalt N., Penadés J.R., Gottesman S. (2021). Molecular Basis of Lysis-Lysogeny Decisions in Gram-Positive Phages. Annual Review of Microbiology.

[21] Casjens S.R., Hendrix R.W. (2015). Bacteriophage lambda: Early pioneer and still relevant. Virology.

[22] Kaper J.B., Nataro J.P., Mobley H.L.T. (2004). Pathogenic *Escherichia coli*. Nat. Rev. Microbiol..

[23] Lin D.M., Koskella B., Lin H.C. (2017). Phage therapy: An alternative to antibiotics in the age of multi-drug resistance. World J. Gastrointest. Pharmacol. Ther..

[24] Shao Q.Y., Trinh J.T., Zeng L.Y. (2019). High-resolution studies of lysis-lysogeny decision-making in bacteriophage lambda. J. Biol. Chem..

[25] Ranquet C., Toussaint A., de Jong H., Maenhaut-Michel G., Geiselmann J. (2005). Control of bacteriophage Mu lysogenic repression. J. Mol. Biol..

[26] Bandyopadhyay K., Parua P.K., Datta A.B., Parrack P. (2010). *Escherichia coli* HflK and HflC can individually inhibit the HflB (FtsH)-mediated proteolysis of λCII in vitro. Arch. Biochem. Biophys..

[27] Oppenheim A.B., Kobiler O., Stavans J., Court D.L., Adhya S. (2005). Switches in bacteriophage lambda development. Annu. Rev. Genet..

[28] Lee S., Lewis D.E.A., Adhya S. (2018). The Developmental Switch in Bacteriophage λ: A Critical Role of the Cro Protein. J. Mol. Biol..

[29] Wang Y.X., Dai J.J., Wang X.H., Wang Y., Tang F. (2022). Mechanisms of interactions between bacteria and bacteriophage mediate by quorum sensing systems. Appl. Microbiol. Biotechnol..

[30] LaSarre B., Federle M.J. (2013). Exploiting Quorum Sensing To Confuse Bacterial Pathogens. Microbiol. Mol. Biol. Rev..

[31] Ramírez-Sánchez I., Magos-Castro M., Guarneros G. (2023). Transcriptional analysis in bacteriophage Fc02 of *Pseudomonas aeruginosa* revealed two overlapping genes with exclusion activity. Front. Microbiol..

[32] Veses-Garcia M., Liu X., Rigden D.J., Kenny J.G., McCarthy A.J., Allison H.E. (2015). Transcriptomic Analysis of Shiga-Toxigenic Bacteriophage Carriage Reveals a Profound Regulatory Effect on Acid Resistance in *Escherichia coli*. Appl. Environ. Microbiol..

[33] Sundarram A., Britton B.C., Liu J., Desiree K., Ogas R., Lemaster P., Navarrete B., Nowakowski H., Harrod M.K., Marks D. (2021). Lytic Capacity Survey of Commercial *Listeria* Phage Against *Listeria* spp. with Varied Genotypic and Phenotypic Characteristics. Foodborne Pathog. Dis..

[34] Taj M.K., Ling J.X., Bing L.L., Qi Z., Taj I., Hassani T.M., Samreen Z., Yunlin W. (2014). Effect of dilution, temperature and pH on the lysis activity of T4 phage against *E. coli* BL21. J. Anim. Plant Sci..

[35] Martín R., Soberón N., Escobedo S., Suárez J.E. (2009). Bacteriophage induction versus vaginal homeostasis: Role of H_2_O_2_ in the selection of *Lactobacillus* defective prophages. Int. Microbiol..

[36] Jiang L., Liu P., Wu T., Guo S. (2020). Genitic information and biological characteristics of mycobacteriophage Chy5. Chin. J. Zoonoses.

[37] Ameh E.M., Tyrrel S., Harris J.A., Pawlett M., Orlova E.V., Ignatiou A., Nocker A. (2020). Lysis Performance of Bacteriophages with Different Plaque Sizes and Comparison of Lysis Kinetics After Simultaneous and Sequential Phage Addition. Phage.

[38] Guillon A., Pardessus J., L’Hostis G., Fevre C., Barc C., Dalloneau E., Jouan Y., Bodier-Montagutelli E., Perez Y., Thorey C. (2021). Inhaled bacteriophage therapy in a porcine model of pneumonia caused by *Pseudomonas aeruginosa* during mechanical ventilation. Br. J. Pharmacol..

[39] Igler C., Abedon S.T. (2019). Commentary: A Host-Produced Quorum-Sensing Autoinducer Controls a Phage Lysis-Lysogeny Decision. Front. Microbiol..

[40] Ingmer H., Gerlach D., Wolz C. (2019). Temperate Phages of *Staphylococcus aureus*. Microbiol. Spectr..

[41] Shapiro J.W., Putonti C. (2020). UPΦ phages, a new group of filamentous phages found in several members of *Enterobacteriales*. Virus Evol..

[42] Mäntynen S., Laanto E., Oksanen H.M., Poranen M.M., Díaz-Muñoz S.L. (2021). Black box of phage-bacterium interactions: Exploring alternative phage infection strategies. Open Biol.

[43] Cenens W., Makumi A., Govers S.K., Lavigne R., Aertsen A. (2015). Viral Transmission Dynamics at Single-Cell Resolution Reveal Transiently Immune Subpopulations Caused by a Carrier State Association. PLoS Genet..

[44] Pinheiro J., Lyons T., Heras V.L., Recio M.V., Gahan C.G.M., O’Sullivan T.P. (2023). Investigation of halogenated furanones as inhibitors of quorum sensing-regulated bioluminescence in *Vibrio harveyi*. Future Med. Chem..

[45] Zhou L.T., Zhang Y., Ge Y.Z., Zhu X., Pan J.Y. (2020). Regulatory Mechanisms and Promising Applications of Quorum Sensing-Inhibiting Agents in Control of Bacterial Biofilm Formation. Front. Microbiol..

[46] Fan Q.Y., Zuo J., Wang H.K., Grenier D., Yi L., Wang Y. (2022). Contribution of quorum sensing to virulence and antibiotic resistance in zoonotic bacteria. Biotechnol. Adv..

[47] Zhang J.J., Feng T., Wang J.Y., Wang Y., Zhang X.H. (2019). The Mechanisms and Applications of Quorum Sensing (QS) and Quorum Quenching (QQ). J. Ocean Univ..

[48] Erez Z., Steinberger-Levy I., Shamir M., Doron S., Stokar-Avihail A., Peleg Y., Melamed S., Leavitt A., Savidor A., Albeck S. (2017). Communication between viruses guides lysis-lysogeny decisions. Nature.

[49] Howard-Varona C., Hargreaves K.R., Abedon S.T., Sullivan M.B. (2017). Lysogeny in nature: Mechanisms, impact and ecology of temperate phages. ISME J..

[50] Bernard C., Li Y.Y., Lopez P., Bapteste E. (2021). Beyond arbitrium: Identification of a second communication system in Bacillus phage phi3T that may regulate host defense mechanisms. ISME J..

[51] Ghosh D., Roy K., Williamson K.E., Srinivasiah S., Wommack K.E., Radosevich M. (2009). Acyl-Homoserine Lactones Can Induce Virus Production in Lysogenic Bacteria: An Alternative Paradigm for Prophage Induction. Appl. Environ. Microbiol..

[52] Jones F.-E. (2000). Inhibition of N-acyl-homoserine Lactone Quorum Sensing.

[53] Jacoby M.L., Hogg G.D., Assaad M.R., Williamson K.E. (2024). Seasonal trends in lysogeny in an Appalachian oak-hickory forest soil. Appl. Environ. Microbiol..

[54] Azimi S., Klementiev A.D., Whiteley M., Diggle S.P., Gottesman S. (2020). Bacterial Quorum Sensing During Infection. Annual Review of Microbiology.

[55] Coolahan M., Whalen K.E. (2025). A review of quorum-sensing and its role in mediating interkingdom interactions in the ocean. Commun. Biol..

[56] Taj M.K., Bing L.L., Qi Z., Ling J.X., Taj I., Hassani T.M., Samreen Z., Mangle A., Wei Y.L. (2014). Quorum Sensing Molecules Acyl-Homoserine Lactones and Indole Effect on T4 Bacteriophage Production and Lysis Activity. Pak. Vet. J..

[57] Laganenka L., Sander T., Lagonenko A., Chen Y., Link H., Sourjik V. (2019). Quorum Sensing and Metabolic State of the Host Control Lysogeny-Lysis Switch of Bacteriophage T1. mBio.

[58] Rossmann F.S., Racek T., Wobser D., Puchalka J., Rabener E.M., Reiger M., Hendrickx A.P.A., Diederich A.K., Jung K., Klein C. (2015). Phage-mediated Dispersal of Biofilm and Distribution of Bacterial Virulence Genes Is Induced by Quorum Sensing. PLoS Pathog..

[59] Hoyland-Kroghsbo N.M., Mærkedahl R.B., Lo Svenningsen S. (2013). A Quorum-Sensing-Induced Bacteriophage Defense Mechanism. mBio.

[60] Wei Z.Y., Li T., Gu Y., Zhang Q., Wang E.H., Li W.B., Wang X., Li Y., Li H.Y. (2022). Design, Synthesis, and Biological Evaluation of N-Acyl-Homoserine Lactone Analogs of Quorum Sensing in *Pseudomonas aeruginosa*. Front. Chem..

[61] Shah M.G., Taylor V.L., Bona D., Tsao Y., Stanley S.Y., Pimentel-Elardo S.M., McCallum M., Bondy-Denomy J., Howell P.L., Nodwell J.R. (2021). A phage-encoded anti-activator inhibits quorum sensing in *Pseudomonas aeruginosa*. Mol. Cell.

[62] Sheriff E.K., Salvato F., Andersen S.E., Chatterjee A., Kleiner M., Duerkop B.A. (2024). Enterococcal quorum-controlled protease alters phage infection. FEMS Microbes.

[63] Hammer B.K., Bassler B.L. (2003). Quorum sensing controls biofilm formation in *Vibrio cholerae*. Mol. Microbiol..

[64] Tan D.M., Hansen M.F., de Carvalho L.N., Roder H.L., Burmolle M., Middelboe M., Svenningsen S.L. (2020). High cell densities favor lysogeny: Induction of an H20 prophage is repressed by quorum sensing and enhances biofilm formation in *Vibrio anguillarum*. ISME J..

[65] Croxatto A., Pride J., Hardman A., Williams P., Cámara M., Milton D.L. (2004). A distinctive dual-channel quorum-sensing system operates in *Vibrio anguillarum*. Mol. Microbiol..

[66] Mauritzen J.J., Sondberg E., Kalatzis P.G., Roager L., Gram L., Svenningsen S.L., Middelboe M. (2023). Strain-specific quorum-sensing responses determine virulence properties in *Vibrio anguillarum*. Environ. Microbiol..

[67] Tan D.M., Lo Svenningsen S., Middelboe M. (2015). Quorum Sensing Determines the Choice of Antiphage Defense Strategy in *Vibrio anguillarum*. mbio.

[68] Bru J.L., Rawson B., Trinh C., Whiteson K., Hoyland-Kroghsbo N.M., Siryaporn A. (2019). PQS Produced by the *Pseudomonas aeruginosa* Stress Response Repels Swarms Away from Bacteriophage and Antibiotics. J. Bacteriol..

[69] Broniewski J.M., Chisnall M.A.W., Hoyland-Kroghsbo N.M., Buckling A., Westra E.R. (2021). The effect of Quorum sensing inhibitors on the evolution of CRISPR-based phage immunity in *Pseudomonas aeruginosa*. ISME J..

[70] Moreau P., Diggle S.P., Friman V.P. (2017). Bacterial cell-to-cell signaling promotes the evolution of resistance to parasitic bacteriophages. Ecol. Evol..

[71] Hoyland-Kroghsbo N.M., Bassler B.L. (2022). Phage Infection Restores PQS Signaling and Enhances Growth of a *Pseudomonas aeruginosa lasI* Quorum-Sensing Mutant. J. Bacteriol..

[72] Hendrix H., Zimmermann-Kogadeeva M., Zimmermann M., Sauer U., De Smet J., Muchez L., Lissens M., Staes I., Voet M., Wagemans J. (2022). Metabolic reprogramming of *Pseudomonas aeruginosa* by phage-based quorum sensing modulation. Cell Rep..

[73] Schwartzkopf C.M., Robinson A.J., Ellenbecker M., Faith D.R., Schmidt A.K., Brooks D.M., Lewerke L., Voronina E., Dandekar A.A., Secor P.R. (2023). Tripartite interactions between filamentous Pf4 bacteriophage, *Pseudomonas aeruginosa*, and bacterivorous nematodes. PLoS Pathog..

[74] Lee J., Zhang L.H. (2015). The hierarchy quorum sensing network in *Pseudomonas aeruginosa*. Protein Cell.

[75] Qin X.Y., Sun Q.H., Yang B.X., Pan X.W., He Y., Yang H.J. (2017). Quorum sensing influences phage infection efficiency via affecting cell population and physiological state. J. Basic Microbiol..

[76] Lavigne R., Lecoutere E., Wagemans J., Cenens W., Aertsen A., Schoofs L., Landuyt B., Paeshuyse J., Scheer M., Schobert M. (2013). A Multifaceted Study of *Pseudomonas aeruginosa* Shutdown by Virulent Podovirus LUZ19. mbio.

[77] Friman V.P., Ghoul M., Molin S., Johansen H.K., Buckling A. (2013). Pseudomonas aeruginosa adaptation to lungs of cystic fibrosis patients leads to lowered resistance to phage and protist enemies. PLoS ONE.

[78] Silpe J.E., Bassler B.L. (2019). A Host-Produced Quorum-Sensing Autoinducer Controls a Phage Lysis-Lysogeny Decision. Cell.

[79] van Kessel J.C., Mukherjee S. (2021). Another battle won in the phage-host arms race: *Pseudomonas* phage blocks quorum sensing regulator LasR. Mol. Cell.

[80] Xiao G.P., He J.X., Rahme L.G. (2006). Mutation analysis of the *Pseudomonas aeruginosa mvfR* and *pqsABCDE* gene promoters demonstrates complex quorum-sensing circuitry. Microbiology.

[81] Xuan G.H., Lin H., Tan L., Zhao G., Wang J.X. (2022). Quorum Sensing Promotes Phage Infection in *Pseudomonas aeruginosa* PAO1. mbio.

[82] Chen Y., Ho J.M.L., Shis D.L., Gupta C., Long J., Wagner D.S., Ott W., Josic K., Bennett M.R. (2018). Tuning the dynamic range of bacterial promoters regulated by ligand-inducible transcription factors. Nat. Commun..

[83] Farrow J.M., Hudson L.L., Wells G., Coleman J.P., Pesci E.C. (2015). CysB Negatively Affects the Transcription of pqsR and Pseudomonas Quinolone Signal Production in *Pseudomonas aeruginosa*. J. Bacteriol..

[84] Papenfort K., Silpe J.E., Schramma K.R., Cong J.P., Seyedsayamdost M.R., Bassler B.L. (2017). A *Vibrio* cholerae autoinducer-receptor pair that controls biofilm formation. Nat. Chem. Biol..

[85] Silpe J.E., Bassler B.L. (2019). Phage-Encoded LuxR-Type Receptors Responsive to Host-Produced Bacterial Quorum-Sensing Autoinducers. mbio.

[86] Gu Y., Zhi S.X., Yang N., Yang W.S. (2021). Understanding the mechanism of asymmetric gene regulation determined by the VqmA of vibriophage. Biochem. Biophys. Res. Commun..

[87] Silpe J.E., Bridges A.A., Huang X.L., Coronado D.R., Duddy O.P., Bassler B.L. (2020). Separating Functions of the Phage-Encoded Quorum-Sensing-Activated Antirepressor Qtip. Cell Host Microbe.

[88] Jung S.A., Hawver L.A., Ng W.L. (2016). Parallel quorum sensing signaling pathways in *Vibrio cholerae*. Curr. Genet..

[89] Watve S., Barrasso K., Jung S.R.A., Davis K.J., Hawver L.A., Khataokar A., Palaganas R.G., Neiditch M.B., Perez L.J., Ng W.L. (2020). Parallel quorum-sensing system in *Vibrio cholerae* prevents signal interference inside the host. PLoS Pathog..

[90] Hargreaves K.R., Kropinski A.M., Clokie M.R.J. (2014). What Does the Talking? Quorum Sensing Signalling Genes Discovered in a Bacteriophage Genome. PLoS ONE.

[91] Duddy O., Bassler B.L. (2021). Quorum Sensing Across Bacterial and Viral Domains. PLoS Pathog..

[92] Eggers C.H., Gray C.M., Preisig A.M., Glenn D.M., Pereira J., Ayers R.W., Alshahrani M., Acabbo C., Becker M.R., Bruenn K.N. (2016). Phage-mediated horizontal gene transfer of both prophage and heterologous DNA by φBB-1, a bacteriophage of *Borrelia burgdorferi*. Pathog. Dis..

[93] León-Félix J., Villicaña C. (2021). The Impact of Quorum Sensing on the Modulation of Phage-Host Interactions. J. Bacteriol..

[94] Shukla A., Parmar P., Rao P., Goswami D., Saraf M. (2020). Twin Peaks: Presenting the Antagonistic Molecular Interplay of Curcumin with LasR and LuxR Quorum Sensing Pathways. Curr. Microbiol..

[95] Rajamanikandan S., Srinivasan P. (2017). Exploring the selectivity of auto-inducer complex with LuxR using molecular docking, mutational studies and molecular dynamics simulations. J. Mol. Struct..

[96] Leblanc C., Caumont-Sarcos A., Comeau A.M., Krisch H.M. (2009). Isolation and genomic characterization of the first phage infecting *Iodobacteria*: φPLPE, a myovirus having a novel set of features. Environ. Microbiol. Rep..

[97] Mayer C., Muras A., Romero M., López M., Tomás M., Otero A. (2018). Multiple Quorum Quenching Enzymes Are Active in the Nosocomial Pathogen *Acinetobacter baumannii* ATCC17978. Front. Cell. Infect. Microbiol..

[98] Dela Ahator S., Sagar S., Zhu M.Y., Wang J.H., Zhang L.H. (2022). Nutrient Availability and Phage Exposure Alter the Quorum-Sensing and CRISPR-Cas-Controlled Population Dynamics of *Pseudomonas aeruginosa*. mSystems.

[99] Pei K., Zhang J., Zou T.T., Liu Z. (2021). AimR Adopts Preexisting Dimer Conformations for Specific Target Recognition in Lysis-Lysogeny Decisions of *Bacillus* Phage phi3T. Biomolecules.

[100] Zamora-Caballero S., Chmielowska C., Quiles-Puchalt N., Brady A., del Sol F.G., Mancheño-Bonillo J., Felipe-Ruiz A., Meijer W.J.J., Penadés J.R., Marina A. (2024). Antagonistic interactions between phage and host factors control arbitrium lysis-lysogeny decision. Nat. Microbiol.

[101] Verdugo-Fuentes A., Gastélum G., Rocha J., de la Torre M. (2020). Multiple and Overlapping Functions of Quorum Sensing Proteins for Cell Specialization in *Bacillus* Species. J. Bacteriol..

[102] del Sol F.G., Penadés J.R., Marina A. (2019). Deciphering the Molecular Mechanism Underpinning Phage Arbitrium Communication Systems. Mol. Cell.

[103] Neiditch M.B., Capodagli G.C., Prehna G., Federle M.J., Bonini N.M. (2017). Genetic and Structural Analyses of RRNPP Intercellular Peptide Signaling of Gram-Positive Bacteria. Annual Review of Genetics.

[104] Dou C., Xiong J., Gu Y.J., Yin K., Wang J.J., Hu Y.H., Zhou D., Fu X.H., Qi S.Q., Zhu X.F. (2018). Structural and functional insights into the regulation of the lysis-lysogeny decision in viral communities. Nat. Microbiol.

[105] Dunny G.M., Berntsson R.P.A. (2016). Enterococcal Sex Pheromones: Evolutionary Pathways to Complex, Two-Signal Systems. J. Bacteriol..

[106] Wang Q., Guan Z.Y., Pei K., Wang J., Liu Z., Yin P., Peng D.H., Zou T.T. (2018). Structural basis of the arbitrium peptide-AimR communication system in the phage lysis-lysogeny decision. Nat. Microbiol.

[107] Stokar-Avihail A., Tal N., Erez Z., Lopatina A., Sorek R. (2019). Widespread Utilization of Peptide Communication in Phages Infecting Soil and Pathogenic Bacteria. Cell Host Microbe.

[108] Guan Z.Y., Pei K., Wang J., Cui Y.Q., Zhu X., Su X., Zhou Y.B., Zhang D.L., Tang C., Yin P. (2019). Structural insights into DNA recognition by AimR of the arbitrium communication system in the SPbeta phage. Cell Discov..

[109] del Sol F.G., Quiles-Puchalt N., Brady A., Penadés J.R., Marina A. (2022). Insights into the mechanism of action of the arbitrium communication system in SPbeta phages. Nat. Commun..

[110] Jiang X.B., Kang R., Yu T., Jiang X.J., Chen H., Zhang Y.P., Li Y., Wang H.L. (2023). Cinnamaldehyde Targets the LytTR DNA-Binding Domain of the Response Regulator AgrA to Attenuate Biofilm Formation of Listeria monocytogenes. Microbiol. Spectr..

[111] Bruce J.B., Lion S., Buckling A., Westra E.R., Gandon S. (2021). Regulation of prophage induction and lysogenization by phage communication systems. Curr. Biol..

[112] Abedon S.T. (2019). Look Who’s Talking: T-Even Phage Lysis Inhibition, the Granddaddy of Virus-Virus Intercellular Communication Research. Viruses.

[113] Wu S.B., Liu J.H., Liu C.J., Yang A.D., Qiao J.J. (2020). Quorum sensing for population-level control of bacteria and potential therapeutic applications. Cell. Mol. Life Sci..

[114] Laganenka L., Sourjik V. (2023). Bacterial Quorum Sensing Signals at the Interdomain Interface. Isr. J. Chem..

[115] Waters C.M., Bassler B.L. (2005). Quorum sensing: Cell-to-cell communication in bacteria. Annual Review of Cell and Developmental Biology.

[116] Miller M.B., Bassler B.L. (2001). Quorum sensing in bacteria. Annu. Rev. Microbiol..

[117] Silpe J.E., Duddy O.P., Bassler B.L. (2022). Natural and synthetic inhibitors of a phage-encoded quorum-sensing receptor affect phage-host dynamics in mixed bacterial communities. Proc. Natl. Acad. Sci. USA.

[118] Housby J.N., Mann N.H. (2009). Phage therapy. Drug Discov. Today.

[119] Kumar A., Yadav A. (2023). Synthetic phage and its application in phage therapy. Prog. Mol. Biol. Transl. Sci..

[120] Liu Y., Yao Z.C., Mao Z.Z., Tang M.R., Chen H.C., Qian C.R., Zeng W.L., Zhou T.L., Wu Q. (2024). Quorum sensing gene *lasR* promotes phage vB_Pae_PLY infection in *Pseudomonas aeruginosa*. BMC Microbiol..

[121] Pires D.P., Melo L.D.R., Azeredo J., Enquist L.W. (2021). Understanding the Complex Phage-Host Interactions in Biofilm Communities. Annual Review of Virology.

[122] Alvarez-Ordóñez A., Coughlan L.M., Briandet R., Cotter P.D., Doyle M.P., McClements D.J. (2019). Biofilms in Food Processing Environments: Challenges and Opportunities. Annual Review of Food Science and Technology.

[123] Chevallereau A., Pons B.J., van Houte S., Westra E.R. (2022). Interactions between bacterial and phage communities in natural environments. Nat. Rev. Microbiol..

